# Driving a decade of change: HIV/AIDS, patents and access to medicines for all

**DOI:** 10.1186/1758-2652-14-15

**Published:** 2011-03-27

**Authors:** Ellen 't Hoen, Jonathan Berger, Alexandra Calmy, Suerie Moon

**Affiliations:** 1 Medicines Patent Pool Initiative, UNITAID Secretariat, Geneva, Switzerland; 2 SECTION27, Braamfontein, Johannesburg, South Africa; 3 HIV Unit, Division of Infectious Disease, Geneva University Hospital, Geneva, Switzerland; 4 Médecins Sans Frontières Campaign for Access to Essential Medicines, Geneva, Switzerland; 5 Sustainability Science Program, Center for International Development, Kennedy School of Government, Harvard University, Cambridge, MA 02138, USA

## Abstract

Since 2000, access to antiretroviral drugs to treat HIV infection has dramatically increased to reach more than five million people in developing countries. Essential to this achievement was the dramatic reduction in antiretroviral prices, a result of global political mobilization that cleared the way for competitive production of generic versions of widely patented medicines.

Global trade rules agreed upon in 1994 required many developing countries to begin offering patents on medicines for the first time. Government and civil society reaction to expected increases in drug prices precipitated a series of events challenging these rules, culminating in the 2001 World Trade Organization's Doha Declaration on the Agreement on Trade-Related Aspects of Intellectual Property Rights and Public Health. The Declaration affirmed that patent rules should be interpreted and implemented to protect public health and to promote access to medicines for all. Since Doha, more than 60 low- and middle-income countries have procured generic versions of patented medicines on a large scale.

Despite these changes, however, a "treatment timebomb" awaits. First, increasing numbers of people need access to newer antiretrovirals, but treatment costs are rising since new ARVs are likely to be more widely patented in developing countries. Second, policy space to produce or import generic versions of patented medicines is shrinking in some developing countries. Third, funding for medicines is falling far short of needs. Expanded use of the existing flexibilities in patent law and new models to address the second wave of the access to medicines crisis are required.

One promising new mechanism is the UNITAID-supported Medicines Patent Pool, which seeks to facilitate access to patents to enable competitive generic medicines production and the development of improved products. Such innovative approaches are possible today due to the previous decade of AIDS activism. However, the Pool is just one of a broad set of policies needed to ensure access to medicines for all; other key measures include sufficient and reliable financing, research and development of new products targeted for use in resource-poor settings, and use of patent law flexibilities. Governments must live up to their obligations to protect access to medicines as a fundamental component of the human right to health.

## Review

### Introduction

A decade ago, the world prepared to gather in Durban, South Africa, for the first International AIDS Conference to be held on the continent most devastated by HIV. At the time, the statistics were grim: only one in a thousand people living with HIV in Africa had access to AIDS treatment [1]. Antiretroviral (ARV) drugs were largely available only from the originator companies that controlled the patents on these medicines, and came with a paralysing price tag of US$10,000 to $15,000 per patient per year [2].

With civil society at the forefront [3-8], a joint mobilization of people living with HIV/AIDS (PLHIV), doctors and nurses, ministries of health, developing country and donor governments [9-12], intergovernmental organizations, and pharmaceutical companies [13,14] achieved today what most delegates at Durban thought impossible: access to ARVs for more than five million people in the developing world [15].

This achievement required some essential ingredients: first, civil society had to put access to treatment for HIV/AIDS on the global political agenda; second, innovative healthcare providers had to demonstrate that delivering treatment was safe and effective and thus feasible in resource-poor settings; and third, the price of medicines had to come down. Once these ingredients were in place, increased funding for ARVs followed, and investment in strengthening health systems to deliver treatment and care for all - both HIV positive and HIV negative - was made possible. Civil society, alongside courageous leaders willing to take risks, made it happen.

While the achievements have been enormous, huge challenges remain to sustain the progress made to date and to meet future needs.

The past 10 years have shown how ARV treatment can reduce HIV/AIDS-related illness and death in developing countries. But in the current climate of wavering support for achieving universal access to treatment, prevention, care and support - a commitment that Member States made at the UN General Assembly just five years ago [16,17] - it is necessary to look ahead to consider how to make an even greater impact.

Overall, ARVs are still underused relative to need, and they still reach people with too much delay. The latest World Health Organization (WHO) guidelines for HIV treatment in resource-poor settings recommend that people should start treatment when their CD4 cell counts are above 350 cells/mm^3 ^rather than 200 [18,19]. Recent guidelines from wealthy countries recommend even earlier initiation of ARVs, at a CD4 cell count of 500 cells/mm^3 ^or above [20]. The WHO recommendation is a critical step toward improving the efficacy of treatment in developing countries, and is also expected to help prevent transmission of the virus [21]. However, it also means that over 14 million people are now in urgent need of treatment, with more than nine million still left empty handed in the waiting room.

In order to address this challenge, ARVs should be more affordable, meet current medical standards, and be developed or adapted for use in the contexts where they are needed: that is, in settings with minimal or no monitoring available (e.g., for toxicity, viral load, or resistance), where refrigeration may be scarce, and where health workers are in short supply.

### Patents and access to medicines

What role do intellectual property rules and practices play in this equation? The AIDS crisis has radically changed conceptions of and policy approaches to patents on medicines. This shift is reflected in changes in international treaties, national law, public policies, and the business practices of pharmaceutical companies. In order to understand current thinking on HIV medicines patents, we need to look back at least to the 1990s.

In 1996, a group of health non-governmental organizations (NGOs) met in Bielefeld, Germany, to discuss the public health implications of new intellectual property rules created by the World Trade Organization (WTO). The Agreement on Trade-Related Aspects of Intellectual Property Rights (TRIPS) was part of the set of treaties that established the WTO in 1994, and had just come into force in 1995. The negotiations leading to TRIPS had been primarily driven by the trade and commercial interests of the industrialized nations [7,8]. While developing country negotiators were able to preserve certain flexibilities in the agreement, such as transition periods for implementation in developing countries, overall, TRIPS was not focused on public health, and civil society organizations were not part of the negotiation process.

TRIPS required that all WTO Members, which today number 153, provide a minimum standard of intellectual property protection, and was enforceable through the WTO dispute settlement procedures. The standards for intellectual property protection that were globally harmonized through the TRIPS Agreement derived primarily from practices in the industrialized countries, where national patent systems had evolved over many years. While proponents argued that TRIPS would increase foreign direct investment, technology transfer and research in the developing countries, critics argued that it would retard industrialization, hamper technology transfer and increase the prices of essential goods, such as medicines and agricultural inputs [4,22-36].

Before TRIPS, pharmaceutical patent policies and practices were diverse. For example, many countries did not consider patents on such products as medicines and food to be in the public interest. Half of the 98 countries that were members of the 1883 Paris Convention on the Protection of Industrial Property (a major international patent treaty prior to TRIPS, now administered by the World Intellectual Property Organization) actively excluded pharmaceutical (product) patenting altogether [37]. Some countries reduced patent terms on medicines, or only made them available for manufacturing processes but not for the end product. Even among the wealthy countries, some did not grant product patents on medicines until relatively recently: for example, Italy and Sweden began granting pharmaceutical patents only in 1978 and Spain in 1992 [38].

TRIPS put an end to this diversity when it required all Members to introduce 20-year patents in all fields of technology; in practice, this requirement meant that many developing countries had to begin offering patents on pharmaceutical products for the first time. Because TRIPS was part of the WTO package, countries that wished to remain Members of the WTO could not opt out of TRIPS or make reservations to the treaty (unlike many other international agreements). The ensuing years saw a wave of intellectual property reforms in most developing countries in response to TRIPS obligations [32]. The policy space that countries once enjoyed to design intellectual property systems in line with their development needs had been dramatically constrained.

In the late 1990s, the potential effect of these new intellectual property rules on access to medicines was little understood, and interest in intellectual property issues among the public health community was still rare.

### The tide begins to turn

In early 1998, 41 drug companies and their representative body sued the new democratic post-apartheid government of South Africa over amendments made in 1997 to its Medicines Act, which aimed to make low-cost medicines more readily available. The companies asserted that it was neither constitutional nor in compliance with the TRIPS Agreement [39].

This lawsuit was brought against the backdrop of the growing AIDS crisis. It came two years after the 1996 International AIDS Conference in Vancouver, Canada, where the world had learned that highly active antiretroviral therapy could transform HIV infection from a disease with a certain death sentence into a chronic, manageable condition. However, while ARVs were becoming available in the industrialized countries, they remained far out of reach of most South Africans and others living in developing countries. At the time, South Africa was (and remains today) home to the largest estimated number of PLHIV in the world.

Big Pharma vs. Nelson Mandela shocked the world's conscience. It was a call to action that pulled many different actors onto the stage.

In 1999, at the United Nations in Geneva, a group of NGOs and AIDS activists held a conference on compulsory licensing for HIV medicines. A compulsory licence is a way to remedy problems caused by a patent, whereby a government body (such as a ministry, court or a statutory tribunal) grants a licence to an entity other than the patent holder, allowing them to produce the patented product in exchange for "adequate remuneration". It is allowed under the TRIPS Agreement, which sets out some procedural requirements but leaves countries free to determine the grounds for issuing a compulsory licence. Industrialized countries have repeatedly used compulsory licensing, including to purchase low-cost medicines. For example, from 1969 until 1992, when Canada changed its system as a requirement of the North American Free Trade Agreement, Canada granted 613 compulsory licences for the production or import of generic medicines, leading to some of the lowest medicines prices in the industrialized world [40]. (A generic drug is a pharmaceutical product, usually intended to be interchangeable with an innovator product.)

Today there is nothing revolutionary or newsworthy about holding a meeting about compulsory licensing and access to medicines, but in 1999, the situation was quite different. Discussing compulsory licensing was the exclusive domain of specialized intellectual property lawyers. The Geneva meeting gathered NGOs and health officials to discuss how flexibilities in intellectual property law, such as compulsory licences, could be used to increase the availability of low-cost HIV medicines in the developing world. This caused a great deal of concern among patent holders.

The growing discontent with the public health implications of TRIPS culminated at the WTO ministerial conference in Seattle in 1999 with a call to "humanize the trade agreements". Advocates from civil society and developing country governments began forming a strong coalition and pushed for the use of measures, such as compulsory licensing, to accelerate the production and availability of low-cost generic medicines for HIV/AIDS, without risk of trade retaliation.

At the time, an editorial in the *Pharmaceutical Executive *commented: "Unlikely as it seems, the pharmaceutical industry may have reason to thank the demonstrators who brought Seattle and the ministerial meeting of the World Trade Organization (WTO) to a standstill. Had the demonstrators not disrupted the gathering, the forecast for global pharma might be much cloudier (Gopal 2000)."

The period between the failed Seattle WTO Ministerial conference in 1999 and the 2001 WTO meeting in Doha saw a number of developments that had a profound effect on intellectual property rules and access to medicines.

Developing countries that were at the forefront of providing ARV therapy began to experience the consequences of pharmaceutical patents on HIV/AIDS drugs. For example, in Thailand and Brazil, patents significantly limited the legal space to produce lower-cost generics, resulting in a heavy burden on public health budgets.

Brazil was the first developing country to provide widespread access to HIV/AIDS treatment through its national programme; the Brazilian programme demonstrated to the world that ARVs could be provided safely even with limited toxicity and efficacy monitoring [41]. Initially, Brazil's programme heavily relied on the ability to produce lower-cost generic versions of ARVs that were not patented in the country. However, like many developing countries, in the 1990s, Brazil had come under strong pressure from wealthy nations to tighten patent protection, and had amended its national laws to begin granting pharmaceutical patents in 1996 (nine years before it was obligated to do so by TRIPS). The high price of patented drugs soon began to consume more and more of the ARV budget. At one point in Brazil, three patented medicines (out of a total of 17) took up 75% of the AIDS programme's drug budget [9,42,43].

At the same time that awareness of the public health implications of TRIPS was growing, the AIDS crisis also began to attract greater political attention at the global level. In 2000, the Group of 8 countries paid unprecedented attention to health and the need for action to increase access to medicines. At the International AIDS Conference in Durban, the Treatment Action Campaign and its partners organized the Global March for Treatment, squarely placing access to ARVs on the political agenda.

In December of that year, a three-day global summit in Okinawa, Japan, on infectious diseases outlined an agenda to prevent the spread of AIDS, provide treatment and care for those affected, and to enhance research and development (R&D) for international public goods, including new approaches to managing intellectual property. Most importantly, Okinawa witnessed the birth of the Global Fund to Fight AIDS, Tuberculosis and Malaria (the Global Fund), a result of extensive efforts by many advocates to create a new approach to financing the international response to HIV and other global health concerns.

Under increasing public pressure to support rather than hinder efforts to combat the epidemic, the patent-holding pharmaceutical industry began to respond. In May 2000, five pharmaceutical companies announced the Accelerating Access Initiative, offering price discounts on HIV-related medicines and diagnostics in developing countries [44]. However, even with the discounts, the prices offered through this initiative paled in comparison with the prices offered by generic producers.

Generic production of ARVs in India was possible because the Indian Patents Act did not provide for patents on pharmaceutical products until required by TRIPS to do so in 2005. Generic producers competed with each other to make medicines at prices far lower than the originators. Indian firms also combined two or more medicines into one pill in "fixed-dose combinations" (FDCs), a type of innovation facilitated by the absence of medicines product patents. FDCs are thought to facilitate patient adherence, reduce the risk of resistance and simplify supply chain management [45-47]. Although Indian firms were not the only ones that produced three-in-one FDCs, they were the first to produce the FDC of stavudine, lamivudine and nevirapine, a first-line regimen recommended by WHO at the time. The convenience for patients and relatively low price of this FDC has helped make it the mainstay of many treatment programmes in developing countries.

In high-income countries, the patents on these three medicines were controlled by three different companies (Bristol Myers Squibb, GlaxoSmithKline and Boehringer Ingelheim), which raised the transaction costs of developing this product. In high-income countries, the first three-in-one FDC comprised of medicines on which patents were controlled by *different *companies was the combination of tenofovir, emtricitabine and efavirenz (brand name Atripla). First approved by the US Food and Drug Administration in 2006, this product has become the standard of care in recent recommendations in high-income countries.

In early 2001, the Indian generic medicines producer, Cipla, offered a triple-combination of ARVs for US$350 per patient per year - or HIV/AIDS treatment for less than a dollar a day [48]. At the time, originator prices through the Accelerating Access Initiative were generally not publicly announced, and eligibility was restricted to a limited list of developing countries [49]. The lowest publicly announced originator price for the same combination of drugs offered by Cipla was about $1000 at the time, but countries negotiated case-by-case with originator companies for price discounts, with wide variation in prices by country [48,50]. In contrast, Cipla publicly offered its price to all countries. Cipla's dramatic price reduction, which received widespread media attention, hammered the message home that many of the multinational drug companies were abusing their market monopoly in the face of a catastrophic human disaster. It also drew attention to the effects of generic competition in bringing drug prices down. India quickly was becoming the "pharmacy of the developing world".

Also in 2001, controversy had broken out over the cost of the drug stavudine (also known as d4T), which came to a head on the Yale University campus in March. Stavudine was developed by researchers at Yale University, which held the patent on the drug. The price of the generic version of stavudine in South Africa was 34 times less than the price of the brand-name product from Bristol Myers Squibb, but the patent prevented its use in South Africa. Under pressure from researchers, students and access advocates, Yale renegotiated its licence with Bristol Myers Squibb to ensure the availability of generic stavudine in developing countries [51,52].

Meanwhile, the Medicines Act court case in South Africa was progressing. In early 2001, an *amicus curiae *brief filed by the AIDS Law Project on behalf of the Treatment Action Campaign put the spotlight on access to ARV treatment and brought the matter to the international stage. In April 2001, after a global public outcry that built on the Treatment Action Campaign's legal intervention and domestic advocacy campaign, the drug companies dropped their case against the South African Government. The landscape had dramatically changed.

Access to medicines and the need to revisit the patent rules that govern them had become part of a larger political agenda, and was no longer the exclusive domain of trade negotiators or intellectual property lawyers.

In November 2001, governments at the WTO Ministerial Conference, in an unprecedented move, adopted the Doha Declaration on TRIPS and Public Health. The Doha Declaration made clear that the TRIPS Agreement "can and should be interpreted and implemented in a manner supportive of WTO Members' right to protect public health and, in particular, to promote access to medicines for all [53]".

This landmark event represented the first significant push back to the relentless march to strengthen private intellectual property rights without regard for societal consequences in poor countries.

### Implementing the Doha Declaration

The 500-word Doha Declaration on TRIPS and Public Health has been essential in making lower-cost generic versions of patented medicines available on a large scale.

In 2003, the WTO adopted the "August 30^th ^decision" in an attempt to find a remedy for legal barriers to exporting sufficient amounts of medicines produced under a compulsory licence, and to ensure that countries that rely on import for their medicines supply could benefit from compulsory licences. Most developing countries do not have domestic manufacturing capacity for ARVs. Although some argued that the absence of ARV patents in a number of African countries meant that intellectual property did not pose a barrier to HIV treatment, this perspective did not take into account the industrial reality that patents in a few producing countries (such as India) could hinder access to generic medicines in scores of importing countries [54,55]. While the solution that was adopted is deeply flawed and should be revised, the proposed TRIPS 31*bis *amendment, which has yet to come into force, is the sole amendment agreed since 1994 not only to TRIPS itself, but to the full set of WTO agreements. Public health concerns in general, and the AIDS crisis in particular, made this happen.

On 1 December 2003, WHO and the Joint United Nations Programme on HIV/AIDS declared the lack of HIV/AIDS treatment to be a global public health emergency and announced the launch of a drive to get three million people on ART by 2005; this was the "3 by 5" campaign. The political momentum of the campaign, combined with newly available funding from governments, the Global Fund and the US President's Emergency Plan for AIDS Relief (PEPFAR), allowed countries to begin purchasing HIV/AIDS medicines in significant volumes.

By 2010, such purchases were predominantly generic drugs [56]. For example, by 2008, 95% (by volume) of the global donor-funded ARV market was comprised of generics, primarily from India [57]. The generic proportion of PEPFAR-purchased ARVs grew from 15% to 89% from 2005 to 2008, with estimated savings to PEPFAR totalling $323 million over the four-year period [58].

How did countries manage the potential barriers posed by patents? While Thailand and Brazil's compulsory licences for ARVs in 2006 and 2007 have been widely publicized, it is perhaps less widely known that over 60 developing countries have procured lower-cost medicines on a large scale using TRIPS flexibilities [4,43,59]. Of these, 17 low- and middle-income countries have issued compulsory licences or government use licences to gain access to generic ARVs, including, most recently, Ecuador in 2010. Twenty-six out of 32 least developed country WTO members authorized importation of generic ARVs with reference to Paragraph 7 of the Doha Declaration, which allowed them to delay granting or enforcing medicines patents until at least 2016 [4]. However, some countries, such as South Africa, have yet to make use of such flexibilities.

In other cases, the policy space for countries to use such flexibilities is being constrained by stringent intellectual property requirements that exceed TRIPS and are contained in bilateral or regional free trade agreements, investment treaties and/or WTO accession agreements [60]. Middle-income developing countries that are seen as potentially lucrative emerging markets, in particular, have been subject to strong bilateral pressure from industrialized countries to refrain from using TRIPS flexibilities. Despite these persistent pressures, however, the use of TRIPS flexibilities to access generic medicines has been widespread and represents a major normative and policy shift from 2000.

Many countries could import generic ARVs, largely because India could produce and export them [57]. There was great concern in the public health community when India had to begin granting pharmaceutical patents in 2005 under its TRIPS obligations. However, the Indian Parliament incorporated public health safeguards in its Patents Act, including strict patentability criteria and the possibility for anyone to oppose the granting of patents. PLHIV supported by the Lawyers Collective used these safeguards successfully to oppose patents on HIV/AIDS medicines that did not fulfill the patentability criteria that India had adopted. A challenge to these provisions by one drug company (Novartis), which did not receive a patent for its cancer drug (Glivec), was rejected [61-63].

Companies have also responded to patent challenges by agreeing to voluntary licences to their patents. For example, GlaxoSmithKline and Boehringer-Ingelheim expanded their voluntary licences in South Africa as part of a settlement reached after the AIDS Law Project, acting on behalf of the Treatment Action Campaign and others, had filed a successful complaint with the South African Competition Commission [64,65]. Companies have also made voluntary licences available in response to the threat of non-voluntary measures, such as compulsory licences and patent oppositions [66]. Such licences are critical because they can encourage robust competition among drug manufacturers; competition drove down first-line regimen prices by 99% over the past decade, from $10,000 to as low as $67 per patient per year [67].

In short, the AIDS crisis has been an engine for change. These changes extend beyond the field of intellectual property and access to medicines, and also include:

• Increasing political attention for global health well beyond HIV and AIDS

• Strengthening the role of civil society in decision making in health policy

• Bringing about new financing mechanisms, such as the Global Fund, PEPFAR and UNITAID, whose beneficiaries go beyond HIV and AIDS

• Catalyzing other innovative approaches to financing development, such as the "Robin Hood tax" [68]

• Expanding healthcare delivery through task shifting from doctors to nurses and/or community health workers [69,70]

• Empowering patients through treatment literacy, and putting PLHIV at the centre of their own treatment

• Catalyzing the establishment of access strategies by the pharmaceutical industry

• Establishing the WHO Prequalification Programme, which helped create the market for low-cost generics by providing quality-assurance and a level playing field for competitors [56]

• Improving the standard of care for chronic conditions in resource-limited settings.

### Changing approaches to R&D

The HIV/AIDS crisis and AIDS activists also impacted the way R&D for new medicines is carried out. Since the 1980s, when the US National Institutes of Health was investing in the development of the first AIDS drugs, PLHIV developed scientific expertise on the virus, clinical trials, research methods and promising candidates for drug development. For example, activists demanded greater freedom to decide which risks they were willing or unwilling to take with experimental therapies, and challenged what they saw as the slow pace of regulatory decisions at the US Food and Drug Administration [71]. In addition, by calling into question the legitimacy of global intellectual property rules and their impact on access to medicines in developing countries, the AIDS crisis also helped to spur new thinking on how to generate R&D that would meet the needs of the world's poor.

A patent can be understood as a type of social contract: in exchange for exclusive rights, patent holders are expected to provide benefits, such as innovation, to society. If, however, these benefits are not forthcoming or not widely available, the contract is not being fulfilled [72]. In the conventional model, R&D priorities are driven primarily by the potential profitability of the market for a medicine. This means that the health needs of those who do not comprise a sufficiently attractive market - because they are too poor or too few - will be neglected.

Between 1975 and 2004, of the 1556 new chemical entities marketed globally, only 20 (1.3%) new drugs were for tropical diseases and tuberculosis, diseases that account for 12% of the total disease burden [73]. In 2006, the WHO Commission on Intellectual Property, Innovation and Public Health concluded that "there is no evidence that the implementation of the TRIPS Agreement in developing countries will significantly boost R&D in pharmaceuticals on Type II and particularly Type III diseases. Insufficient market incentives are the decisive factor [74]." (Type II diseases are incident in both rich and poor countries, but with a substantial proportion of the cases in poor countries. Type III diseases are those that are overwhelmingly or exclusively incident in developing countries.)

A number of new initiatives have been launched to address the problem of insufficient research into the neglected diseases. These include more than two dozen public-private product development partnerships, such as the Drugs for Neglected Diseases initiative [75] and a "priority review voucher" from the US Food and Drug Administration, awarded for the development of a new pharmaceutical for a neglected tropical disease (the voucher can be applied to any new drug application to speed up regulatory review time) [76,77]. At the global level, two years of intergovernmental negotiations culminated in the 2008 Global Strategy and Plan of Action on Public Health, Innovation and Intellectual Property, adopted at the 2008 World Health Assembly [78].

The search is on for new ways to generate needs-driven medical innovation that will meet the needs of both the world's rich and poor. Indeed, the crisis in innovation is not limited to developing countries or neglected diseases alone. While globally, the level of patent protection has increased over the past 20 years, the rate of pharmaceutical innovation has fallen, with an increasing number of "me-too drugs" of little or no therapeutic gain. *Prescrire International *found that 68% of the 3096 new products approved in France between 1981 and 2004 offered "nothing new" over previously available medicines. Furthermore, an analysis of more than 1000 new drugs approved by the US FDA between 1989 and 2000 found that more than three-fourths have no therapeutic benefit over existing products [79].

While patents can provide incentives for innovation if sufficient market prospects exist, granting too many intellectual property rights may also impede rather than accelerate innovation by creating a "tragedy of the anti-commons" [80,81]. At the same time, the high prices of medicines that result from the current innovation system raise ongoing access barriers and serious ethical concerns.

Furthermore, recent improvements in access to first-line ARVs should not mask the need for additional research in this field. The gold standard three-in-one FDC (of tenofovir, emtricitabine and efavirenz) still cannot be used during early pregnancy because of the potential first trimester teratogenicity of efavirenz. In addition, the current widely used regimen (which is nevirapine-based) is not suitable for treatment in the early stages of HIV infection due to increased toxicity.

While tuberculosis (TB) remains the most frequent opportunistic infection of HIV/AIDS, using ARVs in combination with TB drugs is still a challenge. Regimens for patients for whom first-line therapy is failing are still expensive, inconvenient and carry side effects and potential interactions with multiple other drugs, making their use impractical. Economic incentives are insufficient for the industry to develop child-friendly drug formulations. Finally, implementing WHO's new recommendations for earlier initiation of ARV therapy in both children and adults will require an expanded drug formulary geared towards addressing a generalized epidemic. Products should ideally be heat stable, require minimal monitoring, and offer simplified dosing and other features that facilitate adherence.

How can we address these interrelated problems of market-driven R&D priority setting, declining innovation and high medicines prices? A number of new proposals have been put on the table, and are being debated and/or pilot tested, including: rewarding innovation based on therapeutic value; prize funds to attract new "solvers" to a problem; guaranteeing markets for end products; open-source collaborative drug discovery; and an R&D treaty [82-88]. While a full discussion of these proposals is beyond the scope of this article, and many cannot be fully evaluated for years to come, it is worth pointing out several lessons from the experience of HIV/AIDS.

First, competitive production of medicines has consistently proven to be the most powerful and reliable way to reduce drug prices to their lowest sustainable levels [67,89]. New innovation models that can "de-link" the market for medicines production from the market for R&D - such that R&D costs do not need to be recuperated through high prices but are rewarded through other mechanisms - hold the promise of helping to address affordability issues [90].

Second, public involvement in and funding for research plays a key role in accelerating scientific progress. Governments need to invest sufficiently in medical R&D. For example, additional funding is needed to conduct further research on promising tenofovir-containing vaginal microbicides to reduce the risk of HIV transmission - a product that offers the important benefit of being woman-initiated and controlled [91].

Third, PLHIV engagement played a central role in overcoming both innovation and access barriers with respect to treatment for HIV/AIDS. New approaches to generating innovation and ensuring widespread access to the fruits of scientific progress should prioritize the engagement of people directly affected by a disease.

### The "treatment timebomb"

With all of the progress of the past decade in scaling up access to ARVs, what is the problem? Unfortunately, the challenges ahead are formidable and many.

First, the cost of treatment is increasing again because new ARVs are likely to be more widely patented in developing countries and thus more expensive. Even with the high patentability standards implemented in India and other countries, some of the new ARVs are likely to be patented. Without production sources, the countries that rely on importation will find it hard to source low-cost medicines. In addition, patents on individual medicines can make it more difficult to develop new FDCs.

Second, increasing numbers of people will need access to new-generation ARVs: an expanded drug formulary is urgently needed. In addition, about two-thirds of people in need of treatment still do not receive first-line medicines today. ARV prices, particularly in some middle-income developing countries, can still put them out of reach of the people who need them. There is wide variation in the voluntary licensing practices of the patent-holding companies, and such licences too often come with limitations that hamper the full effect of generic competition and the ability to develop FDCs.

Third, advances in research on newer drugs and combinations need to be available worldwide. For example, tenofovir is a promising newer drug that is finally becoming available in resource-limited settings, but experience on how to use it without monitoring or in specific populations (e.g., people with renal damage) is lacking. To avert such situations, research should be carried out in the specific contexts, and taking into account the specific co-morbidities of the target populations where medicines are needed. Some drugs, such as raltegravir, elvitegravir or rilpivirine, are promising, but long-term follow up regarding adverse events is lacking, and the feasibility of their use for treating TB co-infected patients is unclear at this stage.

Fourth, the policy space to produce or import generic versions of patented medicines is shrinking in some developing countries. Stringent intellectual property provisions exceeding TRIPS requirements ("TRIPS-plus") have been negotiated into free trade agreements between industrialized and developing countries, and/or investment and WTO accession agreements. Measures, such as patent term extensions, data exclusivity, patent-registration linkage and border enforcement requirements, can all delay access to generics by lengthening, strengthening or broadening monopolies on medicines [60,92-94]. In addition, some agreements contain measures that confuse legitimate generics with counterfeit medicines; such policies can undermine public health by restricting access to affordable, quality-assured generic medicines [95-99]. Countries that enter into agreements that undermine access to medicines are arguably violating their international human rights obligations [100,101].

Fifth, we are faced with a serious financial crisis that risks setting back the treatment achievements of the past 10 years.

In July 2009, the United Kingdom All Party Parliamentary Group on AIDS called this situation a "treatment timebomb" and called for "political activism" to "ensure that the next generation of drugs is available to the world's poorest in future [102]".

### New approaches to managing intellectual property: the Medicines Patent Pool

We need to go further than where we are today. We need expanded use of the existing flexibilities in patent law and new models to address the second wave of the access crisis. Without generic competition, prices for newer drugs will not come down the same way that they did for the first generation of medicines. One promising new mechanism is the Medicines Patent Pool, established with the support of UNITAID.

UNITAID is a new financing mechanism based on a small solidarity levy on airline tickets, and is supported by 29 countries, the Bill & Melinda Gates Foundation, NGOs and communities. Its mission is to increase access to treatment for HIV/AIDS, TB and malaria by making markets work better for health. UNITAID has raised approximately US$1.5 billion, and seeks to be innovative in the way that it both raises and spends funds [103,104].

It is UNITAID's overarching principle to make markets work better for health that made it a natural birthplace for the Medicines Patent Pool Initiative, which became operational in mid-2010. The idea for an HIV medicines patent pool was first launched at the 2002 International AIDS Conference in Barcelona, Spain, by James Love from Knowledge Ecology International. He had studied the US airplane patent pool that was established in 1917 by the US Government to overcome patent barriers to the mass production of airplanes needed for the military [105]. He suggested doing the same for HIV medicines patents.

The Medicines Patent Pool is a response to the changed global intellectual property environment in which medicines are being more widely patented in developing countries. It is built on the principle of relying on market competition to bring medicines prices down. However, robust competition is possible only if licences are available.

The Pool is expected to work as follows:

Patent holders will make licences available through the Pool that will allow others to produce low-cost generic versions of patented ARVs for use in developing countries. It will be important that the licences cover as many developing countries as possible, both to maximize public health benefit and to ensure economies of scale in generic drug production. The licences are also intended to facilitate the development of FDCs and other formulations adapted for use in resource-poor settings, such as special formulations for treating children, by ensuring that patents do not block generic companies or product development initiatives from carrying out follow-on R&D.

Companies that receive licences from the pool will pay royalties on their sales to the patent holders. The Pool will be a systematic and predictable way of making voluntary licences available, offering legal certainty to all parties involved. No change in international or national law is required for the Pool to work; what is required is a change in mindset from the patent holders, without whose collaboration this initiative cannot succeed. In other words, the Patent Pool will work only if patent holders are willing to collaborate to make their intellectual property available to the Pool. Several major leading patent holders have expressed an interest and willingness to engage with the Pool.

In addition, companies have increasingly adopted voluntary licensing practices as part of their access policies; voluntary measures, such as the Pool, may provide an attractive alternative to non-voluntary measures for patent holders, and can be understood as one outcome of the decade-long evolution in approaches to managing intellectual property and access to medicines. In September 2010, the US National Institutes of Health became the first patent holder to licence its patents (related to a class of ARVs) to the newly established Medicines Patent Pool [106].

Despite these recent developments, the Pool faces many challenges and many key factors have yet to be determined [107-111]. Nevertheless, it provides a clear illustration of the considerable normative shift that has taken place regarding how intellectual property should be handled relative to access to medicines and the central role played by the AIDS crisis in driving forward these debates.

## Conclusions

New approaches to achieving innovation and access to medicines are possible today because of the previous decade of activism that demanded a change in the way we approach intellectual property and public health. The political and civil society mobilization catalyzed by HIV/AIDS was at the forefront of these changes. But three warnings merit attention at this point.

First, initiatives such as the Pool are only one approach to addressing access issues, and must be seen as complements to a broad set of other policies that are needed to ensure access to medicines for all. The Pool is not a panacea, and governments must live up to their responsibilities to protect the health of their populations.

Second, overcoming intellectual property barriers to innovation and competitive production is critical, but is only one piece of the complex machinery required to ensure that we achieve our shared objective of universal access to treatment, care and prevention services for HIV/AIDS. Improving access to medicines also requires addressing regulatory issues, strengthening procurement and supply chains, and establishing pharmacovigilence systems, among other measures. In particular, sufficient levels of funding are critical. Without a market for even the lowest-cost medicines, we cannot expect that anyone will be ready to develop and produce these products.

Third, while there may be progress in key aspects of HIV treatment, needs for the development of new products (such as microbicides) and access to medicines for other diseases remain immense. For example, treatment is often unavailable in many developing countries for both acute infectious diseases and chronic diseases, such as diabetes and cancer [112-114]. Progress against one disease should not allow us to be complacent, nor should it overshadow the scale of ongoing unmet needs.

The struggle for improved access to medicines has been and will be a continuous fight, sometimes an uphill battle, and not always easy to win. But the lessons of the past 10 years show what can be achieved if we mobilize.

We are at a crucial point in time: not only do we need to protect what has been achieved, but we also need to be ambitious and go further. It is feasible that with better-adapted, more affordable ARVs, we can double or triple the number of people on treatment without doubling or tripling the cost. We can also ensure that people have access to better and better-tolerated medicines.

High prices simply cannot be legitimate grounds for withholding lifesaving treatment from people. Access to medicines is a fundamental human right [100,115], which puts the obligation on all of us to do all we can to ensure that it is fully realized.

## List of abbreviations

ARV: antiretroviral; FDC: fixed-dose combination; NGO: non-governmental organization; PEPFAR: US President's Emergency Plan for AIDS Relief; PLHIV: people living with HIV/AIDS; R&D: research and development; TB: tuberculosis; The Global Fund: The Global Fund to Fight AIDS, Tuberculosis and Malaria; TRIPS: Agreement on Trade-Related Aspects of Intellectual Property Rights; WHO: World Health Organization; WTO: World Trade Organization

## Competing interests

EtH is the Executive Director of the Medicines Patent Pool and a former employee of UNITAID. SM is a consultant for the Medicines Patent Pool and has been a consultant for UNITAID.

## Authors' contributions

EtH was involved in the conception and design of the study, analysis and interpretation of data, drafting the manuscript, and revising it critically for important intellectual content. JB was involved in drafting the manuscript and revising it critically for important intellectual content. AC was involved in drafting the manuscript and revising it critically for important intellectual content. SM was involved in the conception and design of the study, analysis and interpretation of data, drafting the manuscript, and revising it critically for important intellectual content. All authors read and approved the final manuscript.
